# Future of Exosome Bioinformatics

**DOI:** 10.14806/ej.28.0.1015

**Published:** 2023-03-07

**Authors:** Dimitrios P. Vlachakis, Kalliopi Io Diakou, George P. Chrousos

**Affiliations:** 1 Laboratory of Genetics, Department of Biotechnology, School of Applied Biology & Biotechnology, Agricultural University of Athens, Athens, Greece; 2 University Research Institute of Maternal and Child Health & Precision Medicine, and UNESCO Chair on Adolescent Health Care, National and Kapodistrian University of Athens, “Aghia Sophia” Children’s Hospital, Athens, Greece; 3 Division of Endocrinology and Metabolism, Center of Clinical, Experimental Surgery and Translational Research, Biomedical Research Foundation of the Academy of Athens, Athens, Greece

Exosomes are under intense study as a promising means for drug or biomarker discovery, primarily due to their implication in intercellular communication and the emergence of disease states and their potential as a cutting-edge, natural delivery system at a nanoscale level. The proteins and nucleic acid cargo of exosomes has been at the center of exosome bioinformatic analysis in the context of health and disease, towards the hunt for novel biomarkers and diagnostics. However, the exosomal lipid composition has been emerging as an interesting target of study as well. Exosomes derived from different sources exhibit enrichment of specific lipid classes and various lipid compositions under different physiological conditions. Therefore, there is a mounting need for exosome lipidomic studies to build the foundation for novel therapeutic studies that use exosomal components. Bioinformatic pipelines are under development to efficiently identify, quantify and elucidate the exosomal lipids and their roles in disease. Cutting-edge bioinformatic tools, such as LipidXplorer, LUX Score, and LipidHome allow the execution of essential analyses such as shotgun lipidomics, and the detection of systematic differences in lipid composition and metadata processing. In the case of pancreatic cancer, an admittedly prevalent and life-threatening disease, these tools have yielded novel exosome lipid biomarkers. Furthermore, bioinformatic platforms such as “Lipidomics Informatics for Life-Science” enable fast and integrated access to these pipelines in a user-friendly manner.

Modern bioinformatic methods facilitate the processing of exosome lipidomics data stemming from mass spectrometry. A set of bioinformatic tools, developed and provided by the LIPID MAPS consortium^1^, allow the prediction of structural components from an input of mass spectrometry data. In addition to the chemical structures, information on the exosomal lipid ontology is readily available. Meta-analysis of the exosomal lipidomics data is also made possible through the platform, which filters the data for non-lipid artifacts and corrects potential errors in the processing of MS spectra before storing the datasets.

Proteins that are secreted by exosomes can have a multitude of effects on the development of pathological conditions. In contrast to eukaryotes, where a signal peptide marks proteins that are to be secreted to allow passage through the ER/Golgi-dependent pathway, the proteins to be secreted by exosomes lack such a signal peptide. Bioinformatic methods can thus bridge the gap toward the effective prediction of protein secretion mediated by exosomes. Intuitive algorithms, such as random forest, can be trained on amino acid sequences of proteins secreted and not secreted by exosomes as a relevant feature, approaching the prediction problem as a classification problem. ExoPred, which implements this method, has been developed to identify proteins secreted by exosomes in vertebrates, marking the potential of cutting-edge algorithmic approaches for predicting and annotating distinct, exosome-secreted proteins and opening new pathways for the subsequent study of their potential role in cell communication.

The study of exosomal miRNAs can lead to discovering valuable information concerning the target mRNAs for these miRNAs. Especially in the context of disease and its progression, the differential expression of exosomal miRNAs represents a source of valuable data that can be analysed with bioinformatic tools. With the increase in computational power, machine learning algorithms are gaining popularity in medicine and exosomal analysis. The strength and adaptability of these methods allow the extraction of relevant and helpful information from the vast array of available databases, which are becoming increasingly open to the public and contain large and diverse biomedical data. The development of powerful algorithms, such as LASSO^2^ enables the extraction of relevant features from a large number of unrelated features. Hence, this modern algorithm can help in the identification of key exosomal miRNAs and mRNAs involved in complex diseases. When combined with adequate cross-validation methods for the strict evaluation of features and parameters, LASSO – and similar feature extraction algorithms – can effectively generate novel information concerning the exosomal RNAs implicated in pathologies.

In cancer screening and precision nanotherapeutics, extracellular vehicles, such as exosomes, from patients are prime candidates for liquid biopsies. Machine intelligence-driven classification methods are rapidly emerging as a solid support system that, when paired with tried-and-true analytical methods, such as time-dependent spectroscopy, can aid the early and accurate detection of malignancies. An assortment of machine learning approaches such as multilayer perceptrons, support vector machines, and AdaBoost random forest classifiers^3^, have been coupled with fluorescence correlation spectroscopy (FCS) conducted on samples of blood-derived vesicles from cancer patients, with the end goal being the accurate classification of tissue specific extracellular vesicles. In parallel, convolutional neural networks (CNNs), quantum CNNs, and networks such as ResNet^4^, show promise as supplementary validation tools when trained on the spectral images generated by the FCS. Along with continuously evolving AI algorithms and refined experimental techniques for exosome characterisation, we believe that the future of exosome bioinformatics is very prominent and its role in precision and personalized medicine will prove invaluable in the years to come.

## Footnotes

1
https://lipidmaps.org/

2
https://corporatefinanceinstitute.com/resources/knowledge/other/lasso/

3
https://medium.com/machine-learning-101/https-medium-com-savanpatel-chapter-6-adaboost-classifier-b945f330af06

4
https://www.mygreatlearning.com/blog/resnet/

